# Metabolomics integrated with transcriptomics reveals the distribution of iridoid and crocin metabolic flux in *Gardenia jasminoides* Ellis

**DOI:** 10.1371/journal.pone.0256802

**Published:** 2021-09-10

**Authors:** Yuan Pan, Xiao Zhao, Yu Wang, Jun Tan, Da-xia Chen

**Affiliations:** 1 Chongqing Academy of Chinese Materia Medica, Chongqing, China; 2 Chongqing Engineering Research Center for Fine Variety Breeding Techniques of Chinese Materia Medica, Chongqing, China; 3 Chongqing Subcenter of National Resource Center for Chinese Materia Medica, China Academy of Chinese Medical Science, Chongqing, China; CSIR-Central Institute of Medicinal and Aromatic Plants, INDIA

## Abstract

*Gardenia jasminoides* Ellis (*G*. *jasminoides*) fruits are used as a resource for obtaining natural colorants and in traditional Chinese herbal medicine. However, *G*. *jasminoides* presents a relatively long flowering period and different ripening periods, so there are significant differences in the accumulation of metabolites in fruits of different colors. In addition, the complete metabolic pathways of iridoidsand crocins, which are used as medicinal composition of *G*. *jasminoides*, are poorly understood at present. In this research, we comprehensively compared the transcriptome and metabolites profiles of the developmental stages and locations of iridoid and crocin biosynthesis. A large number of differentially expressed genes (DEGs) and differentially accumulated metabolites (DAMs) were detected in four groups of samples, and clear variation in the pattern of metabolite abundance and gene expression were observed among different fruit colors and parts. Geniposide and gardenoside mainly accumulated in the sarcocarp of green fruit (GFS) and the sarcocarp of red fruit (FS), respectively. Crocin mainly accumulated in the peel and sarcocarp of red fruits. In the iridoid pathway, we hypothesized that there was a transport mechanism from the sarcocarp to the peel of *G*. *jasminoides* because of the inconsistent expression of *G8O*, *10-HGO* and *IS* associated with differences in fruit ripening. *UGTs* play an important role in the biosynthesis of the active components of *G*. *jasminoides*. Combined transcriptome and metabonomics analysis showed a negative correlation between the biosynthesis of geniposide and crocin. The redirection of the metabolic flux and the regulation of key enzymes may be the main reasons for the changes in the biosynthesis of iridoid and crocin in *G*. *jasminoides* fruit. Our study expended valuable information for functional genomic library and provided new insights for metabolic engineering of secondary metabolite in *G*. *Jasminoides*.

## Introduction

*Gardenia jasminoides* Ellis (*G*. *jasminoides*), belonging to the Rubiaceae family, is an evergreen shrub that is cultivated in multiple areas in China and is adapted to many temperate regions [1]. It has been used for many years to obtain a natural yellow dye [2, 3], and it’s mature and dried fruits are employed in traditional Chinese medicine. Its fruits exhibit a cold and bitter taste, present anti-inflammatory [4], antidiabetic [5], antidepression [6], hepatocyte-protective [7], antioxidant properties [8] and improve the quality of sleep [1, 9]. Iridoids, organic acids, flavonoids and triterpenes have been identified in the fruit of *G*. *jasminoides* [1]. Many chemicals have been isolated and characterized in the fruit, such as geniposide, genipin, gardenoside, crocin and other iridiods. Geniposide is recorded as the quality control standard for *G*. *jasminoides* in the Chinese Pharmacopoeia (2000–2015 edition) [10, 11]. Because of the pharmacological benefits of crocins and their abundance, *G*. *jasminoides* crocins have attracted increasing attention [12]. Research on bioactive constituent synthesis with a focus on *G*. *jasminoides* has gradually increased. Iridoid are cyclopentapyranoid monoterpenoids that exist in many medicinal plants and are still expanding. Iridoid was derived from terpenoids, which was synthesized upstream of the cytosolic mevalonate (MVA) and plastidic 2-C-methyl-D-erythritol 4-phosphate (MEP) pathways, providing precursors of isoprene pyrophosphate (IPP) and dimethylallyl pyrophosphate (DMAPP) for the biosynthesis of iridoid glycosides [13]. Iridoid biosynthesis is initiated from gernyl pyrophosphate, which is converted to secologanin by a series of reactions that include oxidation, reduction, glycosylatios and methylation steps [14]. The pathway is further modified to produce various subclasses of iridoid by different classes of enzymes. The functional analysis of medicinal plants has identified a set of genes that encode key components of the terpenoid and iridoid biosynthesis pathway and its constituent enzymes, such as 1-deoxy-D-xylulose-5-phosphate synthase (DXS); 2-C-methyl-D-erythritol-4-phosphate reductoisomerase (DXR); isopentenyl diphosphate isomerase (IPPI); 4-(cytidyl-5-diphospho)-2-C-methyl-D-erythritol transferase (CMS); 2-phospho-4-(cytidine 5’-diphospho)-2-C-methyl-D-erythritol kinase (CMK); 4-hydroxy-3-methyl-2-enbutenyl-4-diphosphate synthase (HDS); 4-hydroxy-3-methyl-2-(E)-butenlyl diphosphate reductase (HDR); geranyl diphosphate synthase (GPPS); and geranylgeranyl pyrophosphate synthase (GGPPS) [15–19]. Transcriptome analysis of *G*. *jasminoides* tissues has successfully identified genes that are involved in terpenoid and iridoid biosynthesis [10, 11, 20]. In recent years, studies on the biosynthetic pathway of crocin have attracted increasing attention because of their high medicinal and commercial value. The crocin biosynthesis pathway in *Crocus sativus L*. includes the upstream methylerythritol phosphate (MEP) pathway from pyruvate/glyceraldehyde 3-phosphate to geranylgeranyl pyrophosphate (GGPP), the midstream carotenoid pathway from GGPP to zeaxanthin, and the downstream crocin pathway from zeaxanthin to crocin [12, 21]. A variety of catalytic enzymes and coding genes are involved in the whole pathway, including phytoene desaturase (PDS), zeta-carotene isomerase (Z-ISO), zeta-carotene desaturase (ZDS), carotenoid isomerase (CRTISO), lycopene-cyclase (LCYB), carotenoid cleavage dioxygenase (CCD), aldehyde dehydrogenase (ALDH) and glucosyltransferases (UGTs) [22–27]. Previous studies showed that the biosynthesis of iridoids and crocins originated from the same precursor pathway. In *G*. *jasminoides*, relevant studies have been carried out on the above substances, but few studies have investigated the integration of the biosynthesis pathways of the two kinds of substances. Additionally, the distribution of bioactive substances in different organs of *G*. *jasminoides* fruit and the expression of related genes are not clear. Recent technical advancements in transcriptome and metabolome analyses have provided effective ways to identify new genes and metabolites and to elucidate complex secondary metabolic bioprocesses in plants [28]. In this study, based on the widely targeted metabolomics approach and RNA-Seq sequencing, we profiled the transcriptome and metabolome changes in the peel and sarcocarp of *G*. *jasminoides* fruit during various ripening stages to investigate the biosynthesis mechanism of iridoid and crocin. This study analyzes the molecular mechanisms of the synthesis and distribution of the active components of *G*. *jasminoides* fruit from a new perspective, which provides a theoretical basis for the subsequent selection of dominant provenances and variety improvement.

## Materials and methods

### Plant materials

My study did not involve human participants, specimens or tissue samples, or vertebrate animals, embryos or tissues:

1. We state clearly that no specific permissions were required for these locations/activities, and provide details on why this is the case. The planting place is a Medicinal herb planting base of Chongqing Academy of Chinese Materia Medica, where we have carried out related research on the cultivation of *Gardenia jasminoides* Ellis.
2. We confirm that the field studies did not involve endangered or protected species.
3. We confirm that the anthors had received approval from Chongqing Academy of Chinese Materia Medica to collect samples from the plants.

**Fig 1:**
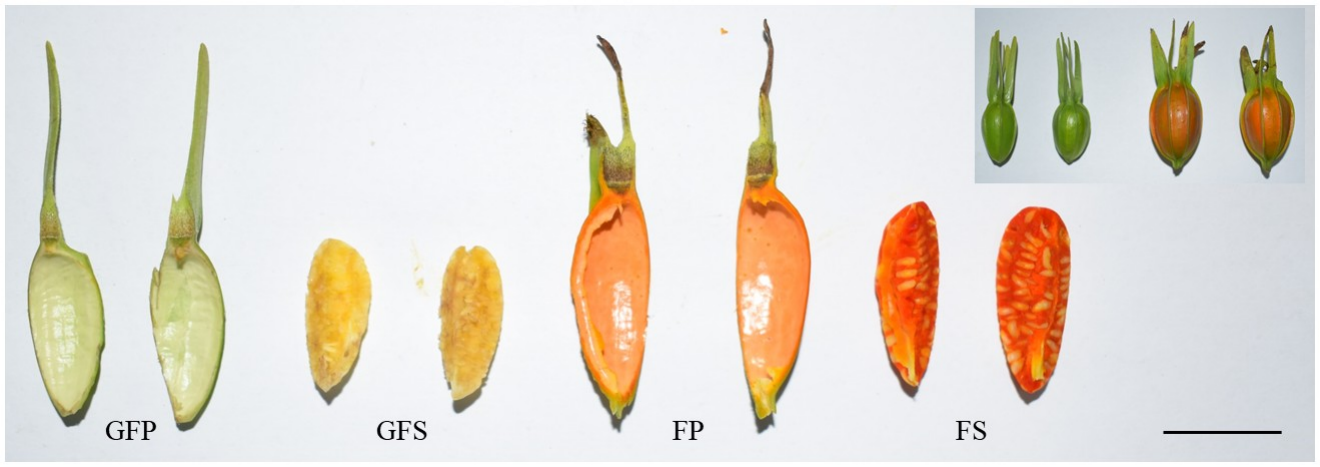
The phenotypes of the *G*. *jasmonoides* fruit during different ripening periods. GFP = the peel of green fruit; GFS = the sarcocarp of green fruit; FP = the peel of red fruit; FS = the sarcocarp of red fruit; Bar = 2 cm.

The widely cultivated *Gardenia jasminoides* Ellis were used for the present experiment, which were presented and identified as *Gardenia jasminoides* Ellis by Professor Daxia Chen. The trees were grown in a Medicinal herb planting base located at the southern hilly region of Banan District, Chongqing, China (N29.34, E106.92). A total of three trees were chosen to collect different fruit samples. All the trees were planted in parallel and were 7 years old. In order to ensure the consistency of sampling, we mark the buds at the same time during the flowering period. The peel of green fruit (GFP), the sarcocarp (the part within the peel of a *G*. *jasmonoides*) of green fruit (GFS), the peel of red fruit (FP) and the sarcocarp of red fruit (FS) were harvested from three plants, respectively (Fig 1). After washing, the samples were immediately frozen in liquid nitrogen and stored at -80°C until further use in metabolite, RNA sequencing (RNA-Seq), and qPCR analyses.

### Metabolite extration

The freezedried samples were ground with zirconia beads for 1.5 min at a 30 Hz stirring mill (MM 400, Retsch, Germany). A total of 100 mg of each powder sample was dissolved in 1.0 mL of the extraction solution (70% methanol solution), which was extracted overnight by a wheel at 4°C. Three vortexes were carried out in this process to ensure complete extraction. Following centrifugation at 10 000×g for 10 min, the extracts were absorbed (CNWBOND Carbon-GCB SPE Cartridge, 250 mg, 3 mL; ANPEL, Shanghai, China, www.anpel.com.cn/cnw) and filtered (SCAA-104, 0.22 μm pore size; ANPEL, Shanghai, China, http://www.anpel.com.cn/) before LC-MS analysis. In addition, quality control samples (mixing) are prepared by mixing all samples in equal amounts. Quality control samples are performed every 10 needles to monitor the stability of the analytical conditions during analysis. The sample preparation, extract analysis, metabolite identification and quantification were performed at Wuhan MetWare Biotechnology Co., Ltd. (www.metware.cn) following their standard procedures and previously fully described by Yuan et al. (2018) and Wang et al. (2017) [29, 30].

### Metabolite analysis by Liquid Chromatography–Electrospray Ionization–Tandem Mass Spectrometry (LC-ESI-MS/MS)

LIT and triple quadrupole (QQQ) scans were acquired on a triple quadrupolelinear ion trap mass spectrometer (Q TRAP), API 4500 Q TRAP LC/MS/MS System, equipped with an ESI Turbo Ion-Spray interface, operating in positive ion mode and controlled by Analyst 1.6.3 software (AB Sciex). The ESI source operation parameters were as follows: ion source, turbo spray; source temperature 550°C; ion spray voltage (IS) 5500 V; ion source gas I (GSI), gas II (GSII), and curtain gas (CUR) were set at 55, 60, and 25.0 psi, respectively; and the collision gas (CAD) was high. Instrument tuning and mass calibration were performed with 10 and 100 μmol/L polypropylene glycol solutions in QQQ and LIT modes, respectively. QQQ scans were acquired as MRM experiments with collision gas (nitrogen) set to 5 psi. DP and CE for individual MRM transitions were performed with further DP and CE optimization. A specific set of MRM transitions was monitored for each period according to the metabolites eluted within this period.

### Qualitative and quantitative analysis

According to previous research results [31, 32], we identified standard substances in self-built databases (MetWare, Wuhan, China) [31] and public databases by comparing fragmentation pattern, retention time and accurate M/Z value. The supervised multivariate method, partial least squares discriminant analysis (PLS-DA), was used to maximize the metabolome differences between a pair of samples. The relative importance of each metabolite to the PLS-DA model was checked using the parameter called variable importance in projection (VIP). Metabolites with VIP ≥ 1 and fold change ≥2 or fold change ≤0.5 were considered differential metabolites for group discrimination [33].

### RNA extraction and transcriptome sequencing

Total RNA was obtained from three different biological replicates for each group using TRIzol reagent (Invitrogen) following the manufacturer’s protocol. Total RNA purity and concentration were determined using the NanoPhotometer^®^ spectrophotometer (IMPLEN, CA, USA) and the Qubit^®^ RNA Assay Kit in Qubit^®^ 2.0 Flurometer (Life Technologies, CA, USA). RNA integrity was assessed using the RNA Nano 6000 Assay Kit of the Agilent Bioanalyzer 2100 system (Agilent Technologies, CA, USA). A total amount of 1.5 μg RNA per sample was used as input material for the RNA sample preparations. Sequencing libraries were generated using NEBNext^®^ Ultra^™^ RNA Library Prep Kit for Illumina^®^ (NEB, USA) following manufacturer’s recommendations and index codes were added to attribute sequences to each sample. The clustering of the index coded samples was performed on a cBot Cluster Generation System using TruSeq PE Cluster Kit v3-cBot-HS (Illumina) according to the manufacturer’s instructions. After cluster generation, the library preparations were sequenced on an Illumina Hiseq 4000 platform and paired-end reads were generated. In the quality control step, raw reads of fastq format were firstly processed through in house perl scripts. In this step, clean reads were obtained by removing reads containing adapter, reads containing ploy-N and low quality reads from raw reads. At the same time, Q20, Q30, GC content and sequence duplication level of the clean data were calculated. All the downstream analyses were based on clean data with high quality. In the transcriptome assembly step, the left files (read1 files) from all libraries/ samples were pooled into one big left.fq file, and right files (read2 files) into one big right.fq file. Transcriptome assembly was accomplished based on the left.fq and right.fq using Trinity [34, 35] with min_kmer_cov set to 2 by default and all other parameters set default. Gene function was performed as previously described [36]: NCBI blast 2.2.28C [37] was used for the alignments of unigenes to Nt database with an E-value threshold of 1E-5. The program diamond (v0.8.22) [38] was selected to perform the comparison against Nr (E-value 1E-5), KOG/COG (E-value 1E-3), and SwissProt (E-value 1E-5) databases. The hmmscan in HMMER 3.0 was operated to search Pfam [39] with an E-value threshold of 0.01. The GO (Gene Ontology) annotations were carried out in Blast2GO (v2.5) [40] with an E-value threshold of 1E-6 based on the Nr and Pfam annotations. Pathway analysis was conducted to elucidate significant pathways of DEGs according to the Kyoto Encyclopedia of Gene and Genomes (KEGG) (http://www.genome.jp/kegg) databases. KOBAS software was used to test the statistical enrichment of differential expression genes in KEGG pathways. The pathways with an FDR value of ≤0.05 were defined as those with genes that display significant levels of differential expression [41].

### Differential expression analysis

The assembled transcriptome spliced by Trinity was used as reference sequence (ref) [42], and the clean reads of each sample were mapped back to this ref in RSEM (v1.2.15) [43] with the Bowtie2 mismatch set 0 as default. The mapping results including read counts of each sample were normalized by calculating the FPKM (expected number of Fragments Per Kilobase of transcript sequence per Millions of base pairs sequenced) to obtain relative expression levels of unigenes [35]. Differential expression analysis of different groups was performed using the DESeq2 R package (v1.6.3). Input data of DESeq2 were clean reads obtained by RSEM. DESeq provide statistical routines for determining differential expression in digital gene expression data using a model based on the negative binomial distribution. The resulting P values were adjusted using the Benjamini and Hochberg’s approach for controlling the false discovery rate. Genes with an adjusted P value (padj) <0.05 and |log2FoldChange| >1 were assigned as differentially expressed.

### Real time quantitative PCR

To analyse DGEs, total RNA was extracted using TRIzol reagent (Invitrogen, CA, USA) from 100 mg of smaples. First strand cDNA was synthesized from 2 μg total RNA using M-MLV Reverse Transcriptase (Promega, WI, USA) according to the manufacturer’s instructions. The reactions (20 μL) were terminated after 40 cycles using the CFX96TM Real Time PCR Detection System (Bio-Rad, CA, USA), and the Actin was used as an internal control. All assays were repeated at least three times. The relevant PCR primer sequences were shown in S1 Table, which were designed based on the CDS sequences of the *G*. *jasmonoides* by the Primer Express Software (Applied Biosystems, CA, USA).

### Correlation analysis between metabolome and transcriptome data

Pearson’s correlation coefficients were calculated between the metabolome and transcriptome data. The coefficients were calculated from log2 (fold change) of each metabolite and log2 (fold change) of each transcript with the Excel program. Correlations with a coefficient of R2 > 0.8 were selected.

### Sequence accession numbers

The whole set of transcript data can be found in the National Center for Biotechnology Information (NCBI) SRA database PRJNA688705.

## Results

### Metabolome characteristics of different colors and parts of *G*. *jasminoides* fruits

**Fig 2:**
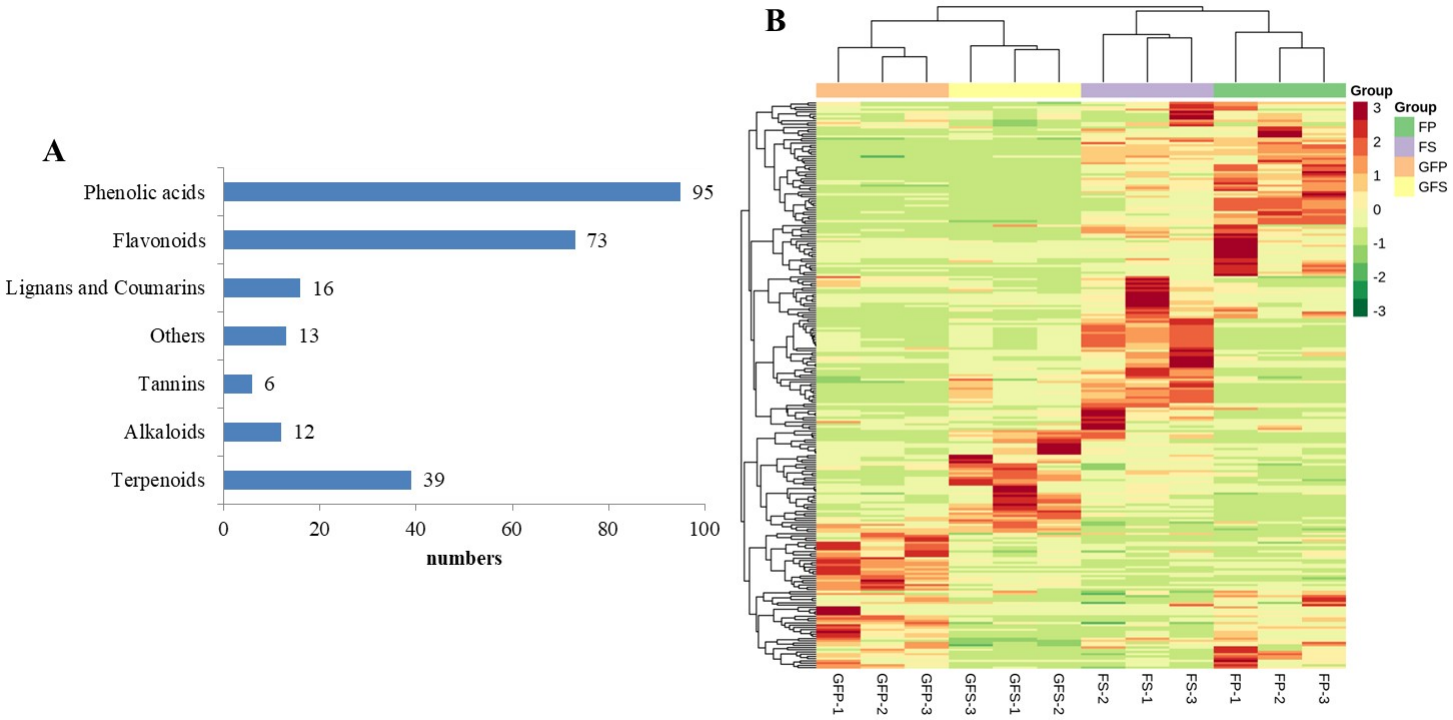
Number and heatmap of metabolite profiles of four groups of samples. (A) Number of different types of metabolites in all samples. (B) Clustering heatmap of the metabolites detected in the total samples. Each example is visualized in a single column and each metabolite is represented by a single row. Red indicates high abundance, whereas low relative metabolites are shown in green. GFP = the peel of green fruit; GFS = the sarcocarp of green fruit; FP = the peel of red fruit; FS = the sarcocarp of red fruit.

Generally, *G*. *jasminoides* blossoms from May to June every year. As the flowers wither, the fruit begins to develop. During the development of *G*. *jasminoides* fruit, the peel changes from green to red, the sarcocarp changes from white to red (Fig 1), and the metabolite composition and contents of the fruit change greatly. The secondary metabolites present in *G*. *jasminoides* during different tissue development stages were investigated based on the widely targeted metabolomics approach by using UPLC-ESI-MS/MS and public and a self-built database (including a MetWare database). A total of 254 secondary metabolites were detected, including 39 terpenoids, 73 flavonoids, 95 phenolic acids, 16 lignans and coumarins, 12 alkaloids, 6 tannins and 13 other metabolites (Fig 2). In the clustering heat map, the accumulation of metabolites displayed clear variation in terms of the pattern of metabolite abundance in different fruit colors and parts, and the green fruit was clearly distinct from the red fruit (Fig 2). We observed that all the biological replicates were grouped together (top side of the figure), indicating high reliability of the metabolome data. In particular, we observed clear separation among GFP, GFS, FP and FS. The above results indicate that there are obvious differences in the metabolic characteristics of the four groups of samples. As shown in Fig 2A, the metabolites detected in the organs of *G*. *jasminoides* were mainly terpenoids, phenolic acids and flavonoids. These metabolites may be associated with many factors, such as nutritional components and antioxidants. The biosynthesis of the active components of *G*. *jasminoides* is mainly centered on the synthesis of terpenoids in the pathway of secondary metabolites.

### Comparison of metabolites produced by the four groups of samples

**Fig 3:**
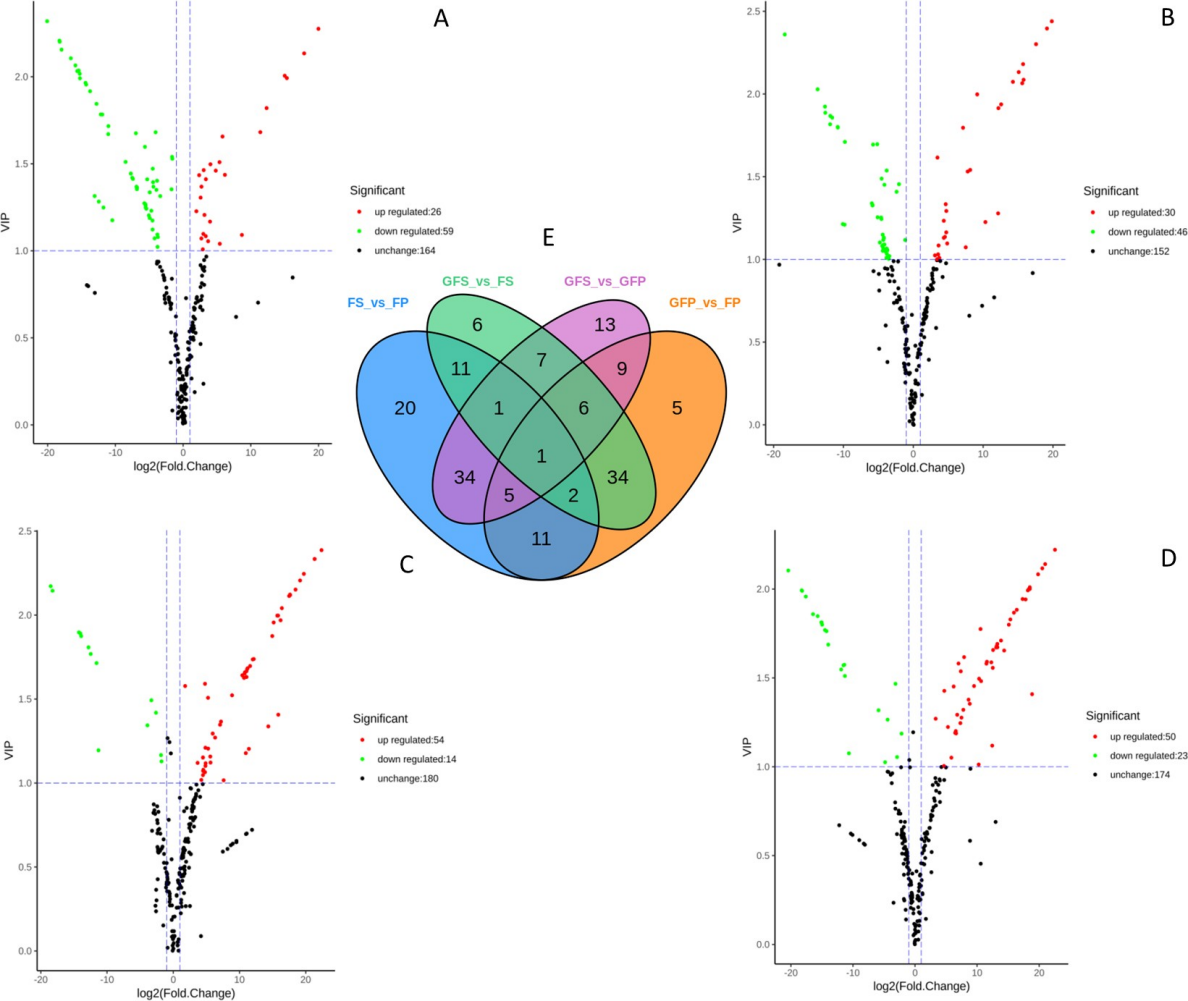
Differential metabolites analysis. Volcano plot of metabolites between FS vs FP (A), GFS vs GFP (B), GFS vs FS (C), GFP vs FP (D); (E): Venn diagram representing numbers of DAMs. GFP = the peel of green fruit; GFS = the sarcocarp of green fruit; FP = the peel of red fruit; FS = the sarcocarp of red fruit. Each point in the volcanic plot represents a metabolite, the abscissa represents the logarithm of the quantitative difference multiples of a metabolite in two samples, and the ordinate represents the variable importance in project (VIP) value. The green dots in the figure represent downregulated differentially expressed metabolites, the red dots represent upregulated differentially expressed metabolites, and the black dots represent metabolites detected but that are not significantly different.

The differentially accumulated metabolites (DAMs) between pairs of samples (GFP vs GFS, GFP vs FP, GFS vs FS and FP vs FS) were screened based on the criterion of a variable importance in projection (VIP) ≥ 1 and a fold change ≥2 or ≤0.5. The DAM screening results are shown in Fig 3. There were 85 kinds of significant DAMs between FS and FP (including 26 downregulated and 59 upregulated compounds in the FS samples) (Fig 3A), 76 kinds of significant DAMs between GFS and GFP (including 30 downregulated and 46 upregulated compounds in the GFS samples) (Fig 3B), 68 kinds of significant DAM between GFS and FS (including 54 downregulated and 14 upregulated compounds in the GFS samples) (Fig 3C), 73 kinds of significant DAM between GFP and FP (including 50 downregulated and 23 upregulated compounds in the GFP samples) (Fig 3D). As seen from the Venn diagram (Fig 3E), there were 11 common DAMs in the four groups of samples, and 76, 73, 68 and 85 specific DAMs were found in GFP vs GFS, GFP vs FP, GFS vs FS and FP vs FS, respectively.

### Transcriptome characteristics of different colors and different parts of *G*. *jasminoides* fruits

In this study, the peel of green fruit (GFP), the sarcocarp of green fruit (GFS), the peel of red fruit (FP) and the sarcocarp of red fruit (FS) of *G*. *jasminoides* were used for transcriptome sequencing. A total of 675 million clean reads were obtained, including 101.2 GB of nucleotide sequence, as shown in Table 1. A total of 52,658 unigene sequences with N50 and N90 lengths of 3,215 bp and 642 bp were obtained by assembling the high quality sequences, as shown in S2 Table. The results showed that the quality of the output data was good and that bioinformatics analysis could be carried out. The unigenes were annotated for gene functions in seven databases, including Nr, Nt, Pfam, KOG/COG, Swissprot, KEGG and GO. A total of 4,729 unigenes were annotated in all databases, accounting for 8.98% of the unigenes, and 35,216 unigenes were annotated in at least one database, accounting for 66.87% of the unigenes (Table 2).

**Table 1:** Summary of sequence analyses.

Sample	Raw Reads	Clean reads	Clean bases	Error(%)	Q20(%)	Q30(%)	GC(%)
GFS1	52071448	51495000	7.72G	0.03	97.53	92.87	43.95
GFS2	56452980	55931656	8.39G	0.03	97.86	93.78	44.06
GFS3	59421610	58623616	8.79G	0.03	98.01	94.14	43.82
GFP1	60246942	59089090	8.86G	0.02	98.05	94.29	44.13
GFP2	60503014	59670552	8.95G	0.03	97.82	93.67	43.89
GFP3	55414260	54316790	8.15G	0.02	98.21	94.65	43.63
FS1	55790180	55255760	8.29G	0.03	97.99	94.07	43.81
FS2	56423132	55609186	8.34G	0.02	98.23	94.65	44.2
FS3	51098270	50416530	7.56G	0.02	98.21	94.56	43.67
FP1	57710694	56340512	8.45G	0.02	98.19	94.61	43.83
FP2	66156372	65204810	9.78G	0.02	98.04	94.23	44.13
FP3	53485158	52782350	7.92G	0.02	98.09	94.32	43.77

**Table 2:** The statistics of gene annotation success rate.

Database	Number of Genes	Percentage (%)
Annotated in NR	30441	57.8
Annotated in NT	19371	36.78
Annotated in KO	12239	23.24
Annotated in SwissProt	23619	44.85
Annotated in PFAM	24169	45.89
Annotated in GO	24169	45.89
Annotated in KOG	8409	15.96
Annotated in all Databases	4729	8.98
Annotated in at least one Database	35216	66.87
Total Unigenes	52658	--

**Fig 4:**
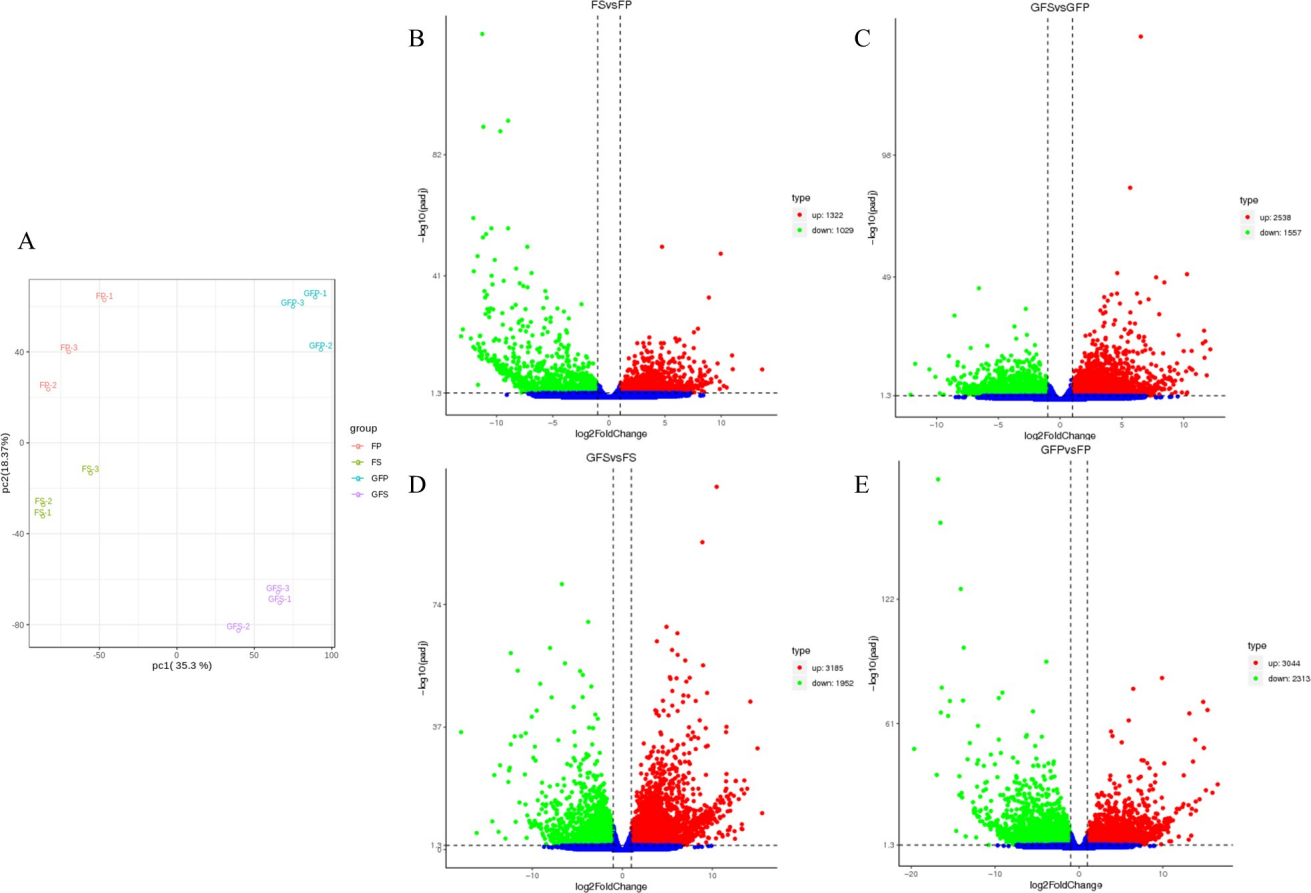
Differential expressed genes (DEGs) in *G*. *jasmonoides* fruits during ripening. (A) Principal component analysis of the twelve samples based on the gene expression profiles; Volcano plots displaying the upregulated, downregulated and noregulated genes between FS vs FP (B), GFS vs GFP (C), GFS vs FS (D), GFP vs FP (E). GFP = the peel of green fruit; GFS = the sarcocarp of green fruit; FP = the peel of red fruit; FS = the sarcocarp of red fruit. The green dots in the figure represent downregulated DEGs, the red dots represent upregulated DEGs, and the blue dots represent genes detected but that are not significantly different.

PCA of the samples based on the fragments per kilobase of exon per million fragments mapped (FPKM) values showed that all the biological replicates clustered together, indicating the high reliability of our results (Fig 4A). According to the results, four groups of samples were clearly distinguished in the PC1 dimension of the PCA score graph (35.3% variation) and were further differentiated in the PC2 dimension, indicating that gene expression varied significantly in different fruit stages and parts. The FPKM method [35] was used to calculate the expression of all unigenes to remove the effects of length differences and sequencing depth. DEGs were defined as genes that were significantly enriched or depleted in one sample relative to another according to DESeq [38] (padj < 0.05 and log2 (fold change) > 1). Compared with FP, 2,351 unigenes in FS showed significant differences; compared with GFP, 4,095 unigenes in GFS showed significant differences; compared with FS, 5,137 unigenes in GFS showed significant changes in their expression levels; and compared with FP, 5,357 unigenes in GFP showed significant differences (Fig 4B–4E).

### KEGG enrichment of DEGs in different groups of samples

**Fig 5:**
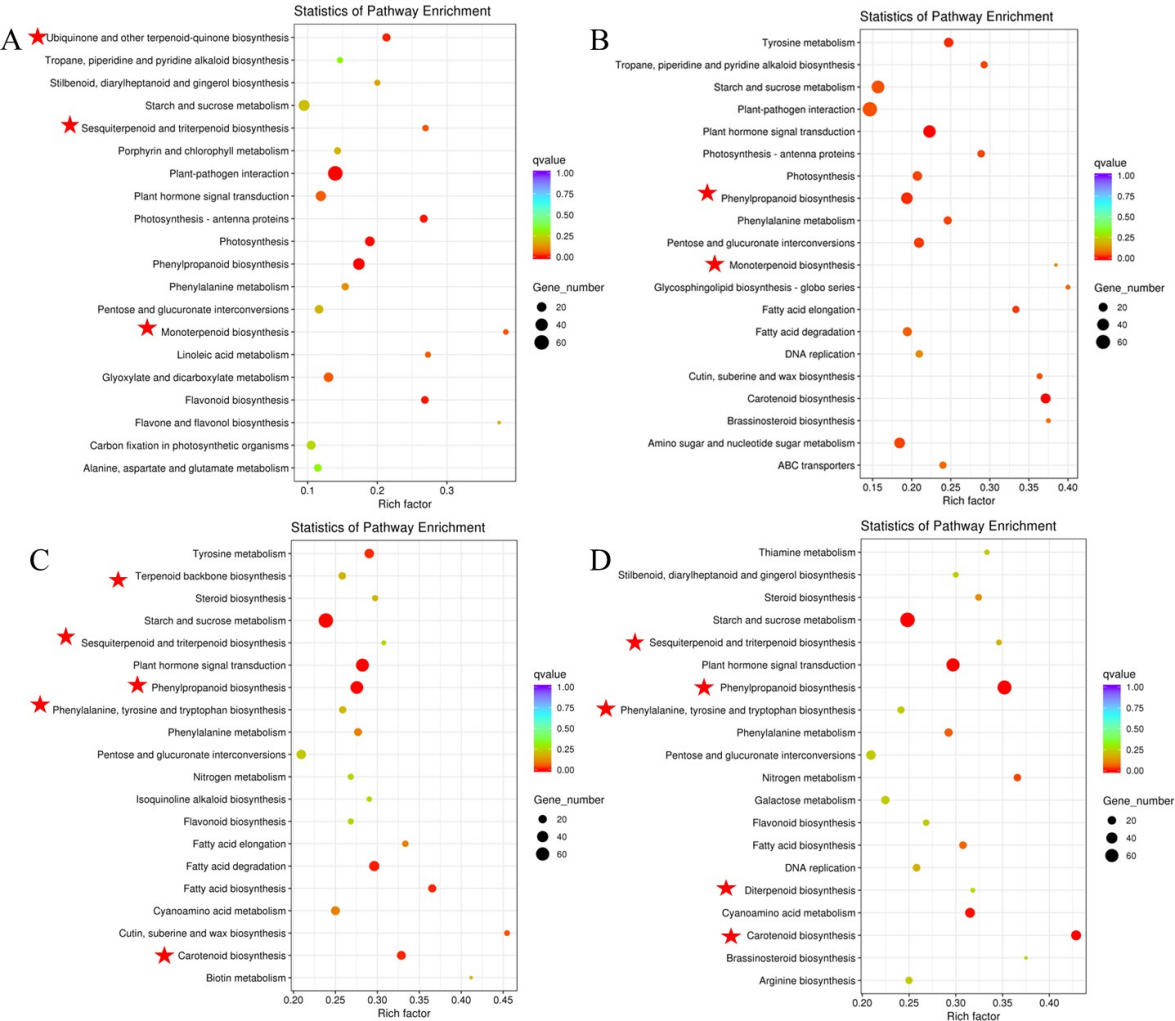
Top 20 KEGG pathway enrichment of DEGs between (A) FS vs FP, (B) GFS vs GFP, (C) GFS vs FS, (D) GFP vs FP. GFP = the peel of green fruit; GFS = the sarcocarp of green fruit; FP = the peel of red fruit; FS = the sarcocarp of red fruit. The dot color represents the qvalue, and the dot size represents the number of differential expressed genes. Red star denotes the metabolic pathway related to the synthesis of iridoids and crocins in *Gardenia jasminoides* Ellis.

KEGG pathway enrichment analysis revealed that these DEGs were mainly enriched in several secondary metabolite metabolism and signal transduction. The results for the top 20 KEGG pathways enriched in each group are represented in scatter plots (Fig 5). According to the enriched pathway of which related to iridoid and crocin biosynthesis (S3 Table), FS vs FP (Fig 5A) mainly included ubiquinone and other terpenoid-quinone biosynthesis, Sesquiterpenoid and triterpenoid biosynthesis, monoterpenoid biosynthesis. GFS vs GFP (Fig 5B) mainly contained phenylpropanoid biosynthesis, monoterpenoid biosynthesis. GFS vs FS (Fig 5C) mainly included phenylpropanoid biosynthesis, carotenoid biosynthesis, terpenoid backbone biosynthesis, phenylalanine, tyrosine and tryptophan biosynthesis, sesquiterpenoid and triterpenoid biosynthesis. GFP vs FP (Fig 5D) mainly concentrated on phenylpropanoid biosynthesis, sesquiterpenoid and triterpenoid biosynthesis, diterpenoid biosynthesis, phenylalanine, diterpenoid biosynthesis, carotenoid biosynthesis. This analysis preliminarily identified obvious differences in the above substances in different developmental stages and parts of fruits and provided basic data for our subsequent research.

### Regulation of MVA/MEP/iridoid pathway genes during fruit development

**Fig 6:**
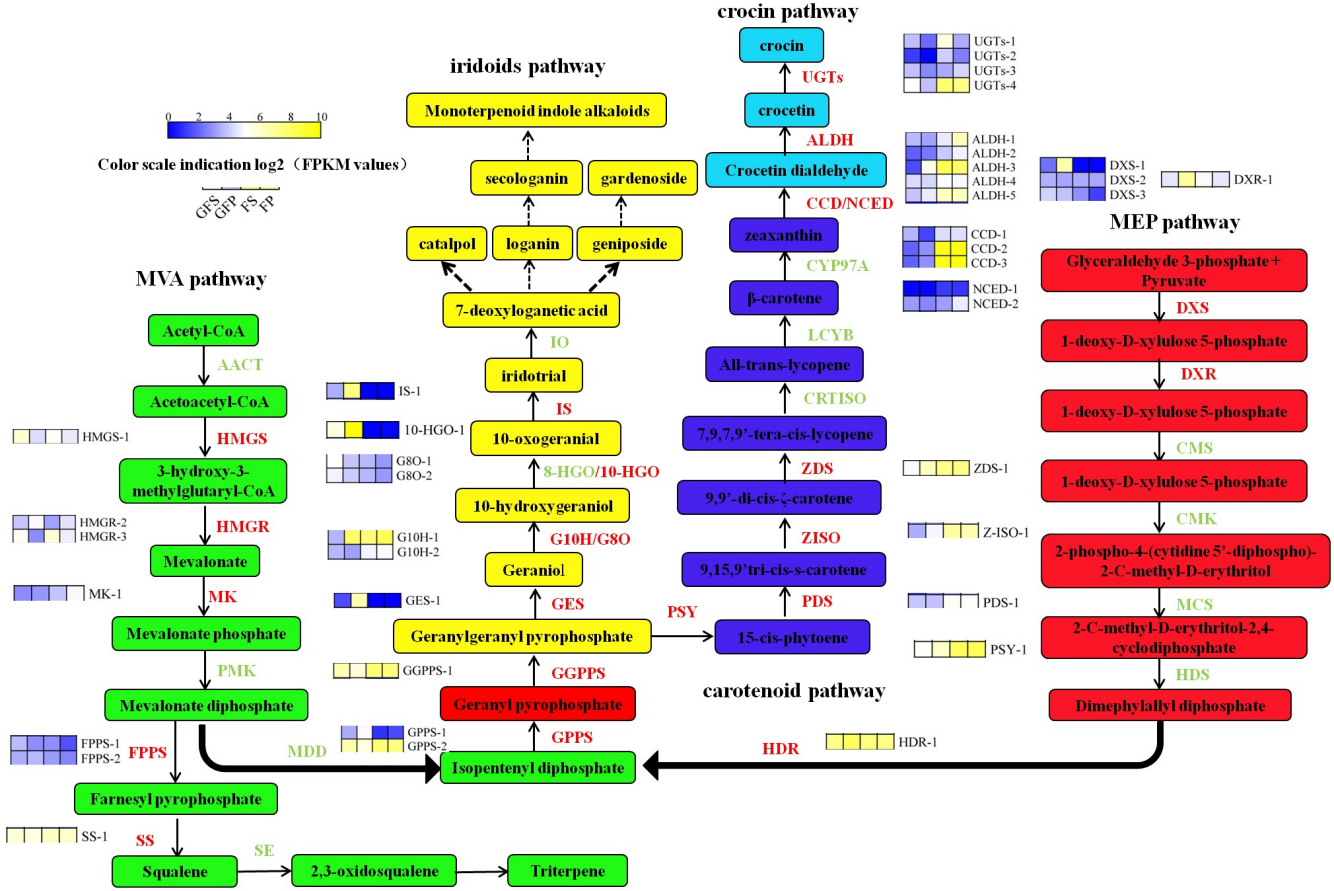
Transcriptional regulations during the *G*. *jasmonoides* fruit development in the biosynthesis of active components. GFP = the peel of green fruit; GFS = the sarcocarp of green fruit; FP = the peel of red fruit; FS = the sarcocarp of red fruit. Gene expression is displayed as heat map depicting the FPKM values. Rectangles marked with yellow and blue background represent increased and reduced expression of genes, respectively. Color scale indication log2 (FPKM values). Multiple transcripts matching each gene are shown side by side near the gene names.

**Fig 7:**
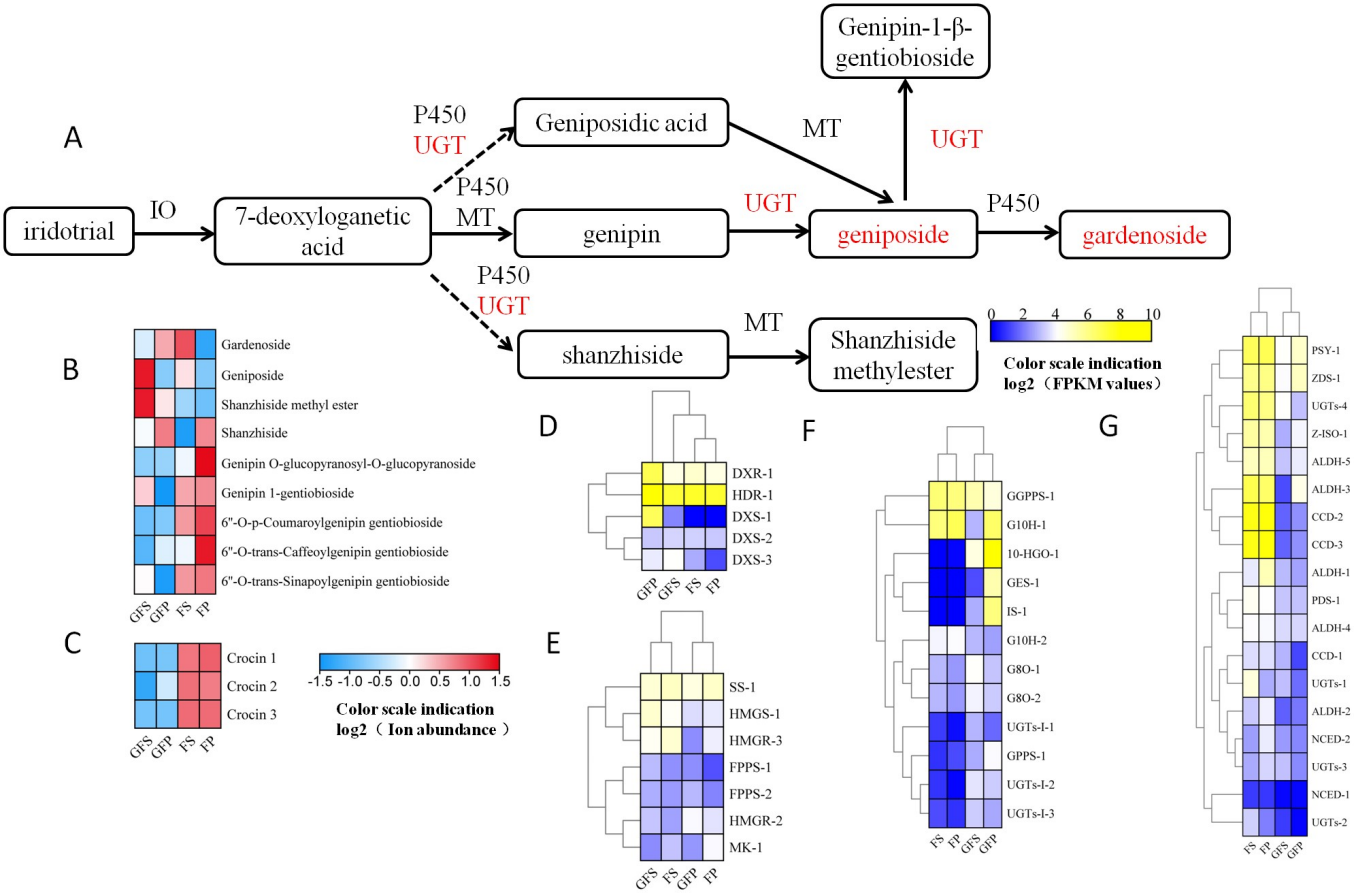
Heatmap and hierarchial clustering showing differentially expressed genes (DEGs) in four pathways. (A) gene regulation during biosynthesis of iridoids; (B) The ion abundance of iridoids in *G*. *jasmonoides*; (C) The ion abundance of crocins in *G*. *Jasmonoides*; (D) DEGs involved in MEP pathway; (E) DEGs involved in MVA pathway; (F)DEGs involved in iridoid pathway; (G) DEGs involved in crocin pathway; GFP = the peel of green fruit; GFS = the sarcocarp of green fruit; FP = the peel of red fruit; FS = the sarcocarp of red fruit. Rectangles marked with red (yellow) and blue background represent increased and reduced expression of genes, respectively.

Previous studies have shown that iridoid are derived from terpenoid, which are synthesized by the upstream MVA and MEP pathways; these pathways produce early precursors of iridoid [10]. In this work, the accumulation rule of iridoid as active components in different maturation stages and different parts of gardenia fruit was studied. This study identified a large number and variety of genes in *G*. *jasminoides* that participate in the biosynthesis of iridoid. Combined with the results of previous studies, we reconstructed the iridoid biosynthesis pathway and obtained candidate unigenes screened from the DEGs. To obtain a systematic view of the iridoid biosynthesis pathway, we observed the abundance of 24 transcripts encoding 15 key enzyme genes involved in iridoid biosynthesis. The whole pathway could be divided into the MAP pathway, MEP pathway and iridoid pathway. As shown in Fig 6, some key enzymes were encoded by more than one unigene. In the MEP pathway, three unigenes were predicted to encode DXS (1-deoxy-D-xylulose-5-phosphate synthase), one unigene was predicted to encode DXR (2-C-methyl-D-erythritol-4-phosphate reductoisomerase), one unigene was predicted to encode HDR (4-hydroxy-3-methyl-2-(E)-butenlyl diphosphate reductase), one unigene was predicted to encode GPPS (geranyl diphosphate synthase) and one unigene was predicted to encode geranylgeranyl diphosphate synthase (GGPPS). We performed cluster analysis of all the differentially expressed genes involved in the MEP pathway and found that the red and green fruits were divided into two groups. According to the change trend of gene expression, 5 genes were divided into two categories: one group showed high expression levels and another group showed low expression levels (Fig 7D).

The key enzymes in the MVA pathway mainly include HMGS (3-hydroxy-3-methylglutaryl CoA synthase), HMGR (3-hydroxy-3-methyl glutaryl CoA reductase), MK (mevalonate kinase), FPPS (farnesyl diphosphate synthase) and SS (squalene synthase) (Fig 6). The same as MEP pathway, a part of genes showed high expression levels and the others showed low levels (Fig 7E). In addition to *SS-1*, *HMGR-1 andHMGR-3*, the expression levels of different predicted genes in the pathway were higher than others. The main enzymes involved in the iridoid pathway were GES (geraniol synthase), G10H (geraniol 10-hydroxylase), G8O (8-hydroxygeraniol dehydrogenase), 10-HGO (10-hydroxygeraniol oxidoreductase) and IS (iridoid synthase). Most genes involved in the iridoid pathway showed an expression pattern similar to those of the genes in the MEP pathway (Figs 6 and 7D–7F). The predicted *DXS-1*, *HDR-1*, *DXR-1*, *G10H-1*, *10-HGO-1*, *GES-1 and IS-1* genes were shown high expression levels in the sarcocarp of green fruit (GFP) compared with their expression in the other three sample types (Fig 6). These expression of predicted genes were consistent with the accumulation of geniposide and are worthy of attention (Fig 7B). Hereafter, we will focus on the genes that were highly expressed in the peel of green fruit (GFP). The opposite expression patterns between the members of certain families indicated the existence of multigene families that may differentially regulate iridoid biosynthesis in *G*. *jasminoides*. The FPKM values of the 24 selected genes are illustrated in S4 Table.

### Key genes involved in crocin biosynthesis were shown significantly high expression level with the development of the fruits

The biosynthesis pathway of crocin in *G*. *jasminoides* is generally decomposed into the MEP pathway, carotenoid pathway and crocin pathway, and iridoid and crocin biosynthesis share the MEP pathway (Fig 6). Eighteen DEGs related to these transcriptomic results were screened as participants in the biosynthesis pathway of crocin (Fig 6). In the carotenoid synthesis pathways, one unigene was predicted to encode phytoene synthase (PSY), one unigene was predicted to encode phytoene desaturase (PDS), one unigene was predicted to encode zeta-carotene isomerase (Z-ISO), and one unigene was predicted to encode zeta-carotene desaturase (ZDS) (Fig 6). The expression of carotenoid isomerase (CRTISO) and lycopene cyclase (LCYB) did not differ significantly in any of the four groups of samples. In addition to *UGTs-1*, the predicted genes showed high expression level in the ripening of fruits. In particular, the expression levels of *PSY-1*, *Z-ISO-1* and *ZDS-1* in the peel of red fruits were significantly higher than those in the sarcocarp. The crocin pathway included four key enzymes: ALDH, CCD, NCED and UGTs (Fig 6). A total of 14 key DEGs were selected in this study, including 3 *CCD*, 2 *NCED*, 5 *ALDH* and 4*UGT* genes. In addition to *NCED-1*, the remaining genes showed an high expression trend in the transformation from green fruit to red fruit. In green fruit, the expression levels of *ALDH-2*, *ALDH-3*, *ALDH-5*, *CCD-2* and *CCD-3* in the peel were higher than those in the sarcocarp. Similarly, most genes in red fruit showed higher expression in the peel than in the sarcocarp (Fig 6). In particular, the FPKM value of *CCD-2* in the red fruit peel was 146.83 times that in green fruit peel and 313.4 times that in green sarcocarp. The metabonomics results indicated that the total ion abundance of crocin-I, crocin-II, and crocin-III in red fruits was higher than that in green fruits, and the abundance in the peel was slightly higher than that in the sarcocarp. The expression trends of ALDH-1, *ALDH-2*, *ALDH-3*, ALDH-4, *ALDH-5*, *UGTs-4*, *CCD-2* and *CCD-3* were similar to the ion abundance trend of crocin, and it is speculated that they are closely related to the biosynthesis of crocin. Among the candidate genes of *CCD*, the homology of *CCD-1* and *CCD-2* with the functional gene *CCD4a* (KY631925.1) which reported by Xu et al. was 84.64% and 99.79%. Among the candidate genes of *UGT*, *UGT-4* had 99.93% homology with *UGT60* (KY631935.1) which reported by Xu et al. Among the candidate genes of *ALDH*, *ALDH-1*, *ALDH-2*, *ALDH-3* have 100.0%, 90.16% and 82.41% homology with *ALDH12* (KY631926.1) which reported by Xu et al., respectively. Especially, the expression trend of the above genes in red or green fruits was similar to Xu et al., which further verified the reliability of transcriptome results (Table 3). The FPKM values of the 18 selected genes are illustrated in S5 Table.

**Table 3:** Candidate genes related to effective constituent biosynthesis.

	Unigene ID	FPKM				Evalue	Identity (%)	Reference sequence ID
		GFS	GFP	FS	FP			
*DXS-1*	Cluster-6394.32347	3.66	102.80	0.09	0.07	0	93.91	XM_027318004.1
*DXS-2*	Cluster-6394.7266	9.97	8.60	9.69	8.79	0	95.31	XM_027228980.1
*DXS-3*	Cluster-6394.8526	16.61	13.13	6.10	1.44	0	96.78	XM_027309974.1
*DXR-1*	Cluster-6394.20265	22.04	114.90	29.02	22.40	3E-177	90.14	JQ038374.1
*HDR-1*	Cluster-6394.18031	154.94	286.80	182.21	164.08	4E-180	80.86	XM_019002578.2
*GPPS-1*	Cluster-6394.3983	9.24	29.24	0.99	1.77	0	85.87	XM_027205307.1
*GGPPS-1*	Cluster-6394.27537	94.79	48.65	203.39	186.09	0	99.91	KY631921.1
*GES-1*	Cluster-6394.3925	1.88	94.14	0.01	0.00	0	94.91	XM_027207804.1
*G10H-1*	Cluster-6394.20184	10.66	249.76	189.49	321.62	0	95.34	XM_027222384.1
*G10H-2*	Cluster-6394.22164	11.22	7.89	27.02	29.95	1E-156	83.77	KF415104.1
*G8O-1*	Cluster-6394.17025	32.42	13.41	11.31	6.92	1E-44	94.17	XM_027323835.1
*G8O-2*	Cluster-6394.26374	26.47	14.98	11.11	7.00	4E-99	93.72	XM_027322805.1
*10-HGO-1*	Cluster-6394.32835	46.06	1026.03	0.00	0.08	2E-18	82.88	KF302069.1
*IS-1*	Cluster-6394.3952	9.70	181.09	0.00	0.00	0	99.47	MN120556.1
*UGTs-I-1*	Cluster-6394.22477	10.71	3.21	1.29	0.18	0	99.50	AB733669.1
*UGTs-I-2*	Cluster-6394.24972	21.40	15.16	1.05	0.06	0	93.33	KY631932.1
*UGTs-I-3*	Cluster-6394.19145	15.10	8.83	1.87	0.98	0	94.61	XM_027236688.1
*HMGS-1*	Cluster-6394.13754	59.55	19.07	34.74	23.33	2E-159	82.51	JF739871.1
*HMGR-2*	Cluster-6394.20158	13.69	28.75	7.92	21.06	0	92.29	XM_027235546.1
*HMGR-3*	Cluster-6394.12073	36.10	5.87	58.09	24.52	2E-171	81.95	U60452.1
*MK-1*	Cluster-6394.19255	5.72	6.70	13.33	28.88	0	93.09	XM_027258792.1
*FPPS-1*	Cluster-6394.28743	11.72	5.94	6.10	2.02	2E-43	100.00	XM_027329076.1
*FPPS-2*	Cluster-6394.7195	9.65	11.27	7.24	5.04	6E-57	86.34	XM_031408199.1
*SS-1*	Cluster-6394.20377	54.71	45.89	71.22	64.04	3E-167	94.18	XM_027304441.1
*PSY-1*	Cluster-6394.20185	32.51	64.45	291.34	327.73	0	93.85	DQ157164.1
*PDS-1*	Cluster-6394.22370	13.77	13.04	35.93	30.92	6E-51	96.03	DQ357179.1
*Z-ISO-1*	Cluster-6394.19655	9.72	27.61	111.71	91.38	0	93.63	XM_027261369.1
*ZDS-1*	Cluster-6394.20056	29.74	72.08	157.36	164.27	3E-12	90.32	LC522877.1
*CCD-1*	Cluster-6394.26865	10.74	1.61	19.29	19.87	0	84.64	KY631925.1
*CCD-2*	Cluster-6394.20229	2.85	6.09	766.37	894.24	0	86.40	XM_027224520.1
*CCD-3*	Cluster-6394.20229	2.85	6.09	766.37	894.24	0	99.79	KY631925.1
*ALDH-1*	Cluster-6394.19872	11.61	8.21	23.17	83.38	6E-72	100.00	KY631926.1
*ALDH-2*	Cluster-6394.21807	2.72	4.09	14.56	25.67	7E-12	90.16	KY631926.1
*ALDH-3*	Cluster-6394.23207	1.83	42.00	348.82	248.85	0	82.41	KY631926.1
*ALDH-4*	Cluster-6394.18648	18.43	17.35	30.55	28.27	0	97.11	XM_027270300.1
*ALDH-5*	Cluster-6394.20156	13.37	23.97	76.29	78.29	0	96.20	XM_027238320.1
*NCED-1*	Cluster-6394.9837	0.11	0.10	1.25	1.12	0	87.89	KY631925.1
*NCED-2*	Cluster-6394.18161	7.13	5.27	7.58	23.73	0	81.40	XM_027315487.1
*UGTs-1*	Cluster-6394.9180	12.68	3.43	48.96	9.60	0	91.10	XM_027263742.1
*UGTs-2*	Cluster-6394.10796	1.28	0.07	16.00	4.65	0	92.54	XM_027309877.1
*UGTs-3*	Cluster-6394.23134	12.78	5.56	9.10	17.10	0	92.25	XM_027248994.1
*UGTs-4*	Cluster-6394.17392	31.67	12.95	215.28	163.24	0	99.93	KY631935.1

### UGT plays an important role in the biosynthesis of the active component of *G*. *jasmonoides*

In *G*. *jasminoides*, *UGTs* are involved in a variety of biosynthesis of secondary metabolite pathways and are also the key downstream enzymes in crocin biosynthesis. In the inferred iridoid pathway (Fig 7A), 7-deoxyloganetic acid generates different iridoid derivatives under the action of various enzymes, and geniposide is finally generated via the action of P450, in which different *UGTs* participate in a multistep reaction. Fig 7A shows that *UGT* participates in geniposidic acid, shanzhiside, geniposide and genipin-1-gentiobioside synthesis. As observed at the transcription level, the expression level of the unigene encoding UGT in GFS was higher than other groups, and the expression level in FP was the lowest. The metabonomic results showed that besides geniposide, Shanzhiside methylester and Shanzhiside, other iridoids presented higher ion abundance in red fruits than in green fruits. Geniposide and Shanzhiside methylester showed higher ion abundance in GFS than other groups, and showed that Shanzhiside in the peel was higher than that in the sarcocarp (Fig 7C, S6 Table). Comprehensive analysis showed that the expression patterns of *UGTS-I-1*, *UGTS-I-2* and *UGTS-I*-3 were positively correlated with the ion abundance of geniposide in sarcocarp (Fig 7B). These results suggested that the three genes were responsible for the biosynthesis of geniposide, and these genes were selected for further analysis. In the crocin pathway, with the exception of *UGT-3*, the remaining *UGTs* were positively correlated with the total ion abundance of the three types of crocin, which was higher in the red fruit than in the green fruit, and that in the sarcocarp was higher than that in the peel (Fig 7C and 7G, S6 Table). We hypothesize that these genes are involved in and regulate the biosynthesis of crocin. In summary, the enzyme activity of *UGTs* directly affects the biosynthesis of iridoids and crocin, and we will conduct further studies on these genes.

### Expression analysis of DEGs by qRT-PCR

To validate the subsequent splicing and assembly and the FPKM expression results, we selected the 51 DEGs for verification of their expression levels by qRT-PCR. Primers were designed in the homologous regions of the unigenes that were transcribed from the same gene, and those with high similarity annotations were retained. 9/51(18%) of candidate gene were found to be inconsistent with respect to expression between the qRT-PCR and RNA-Seq data. Besides deleted genes, t the relative expression levels of the 42 genes in four groups of samples were shown correlations with the Illumina analysis (S1 Fig). The results were within the normal range and the transcriptome results were accurate and efficient.

### Conjoint analysis of DEGs and DAMs associated with other metabolic pathways in four samples

According to the combined analysis of DAMs and DEGs in this study, the different genes and different metabolites in the same group were simultaneously mapped to the KEGG pathway map to better understand the relationships between the genes and metabolites. Table 4 shows that the six groups were also associated with seven metabolic pathways, which included phenylpropanoid biosynthesis, flavonoid biosynthesis, flavone and flavonol biosynthesis, ubiquinone and other terpenoidquinone biosynthesis, tyrosine metabolism, folate biosynthesis and isoquinoline alkaloid biosynthesis. In each group, the numbers of DEGs and DAMs that were enriched in the phenylalanine biosynthesis pathway were greatest. The major DAMs were concentrated in the p-coumaryl alcohol, coniferyl alcohol, scopoletin, 4-hydroxybenzoic acid, syringin and syringetin pathways. These results showed that in addition to the iridoids and crocins present in *G*. *jasminoides* fruit, other DAMs also showed significant differences in different fruit parts and samples from different maturation stages, which also provided basic data for our study on the biosynthesis of other metabolites in *G*. *jasminoides*. Additionally, upstream of the MEP and MVA pathways, the significant DAMs and DEGs identified from the phenylalanine biosynthesis pathway also indicate new research directions.

**Table 4:** DEGs and DAMs involved in other metabolic pathways.

Group	Pathway	Ko ID	Gene Count	Metabolites
GFS vs GFP	Phenylpropanoid biosynthesis	ko00940	38	p-Coumaryl alcohol; Coniferyl alcohol; Scopoletin; Coniferin
	Flavonoid biosynthesis	ko00941	6	(-)-Epicatechin
	Flavone and flavonol biosynthesis	ko00944	1	3,7-Di-O-methylquercetin
GFS vs FS	Ubiquinone and other terpenoid-quinone biosynthesis	ko00130	12	p-Coumaric acid; 4-Hydroxybenzoic acid
	Tyrosine metabolism	ko00350	27	p-Coumaric acid
	Folate biosynthesis	ko00790	3	4-Hydroxybenzoic acid
	Phenylpropanoid biosynthesis	ko00940	54	Syringin; Sinapic acid; Sinapyl alcohol; p-Coumaryl alcohol; Coniferin
	Isoquinoline alkaloid biosynthesis	ko00950	9	Syringin
GFS vs FP	Ubiquinone and other terpenoid-quinone biosynthesis	ko00130	20	4-Hydroxybenzoic acid
	Folate biosynthesis	ko00790	3	4-Hydroxybenzoic acid
	Phenylpropanoid biosynthesis	ko00940	63	p-Coumaryl alcohol; Scopoletin; Caffeic aldehyde; Sinapyl alcohol; Syringin; Coniferin
	Flavonoid biosynthesis	ko00941	15	L-Epicatechin
	Flavone and flavonol biosynthesis	ko00944	4	3,7-Di-O-methylquercetin; Syringetin
GFP vs FS	Ubiquinone and other terpenoid-quinone biosynthesis	ko00130	12	p-Coumaric acid; 4-Hydroxybenzoic acid
	Tyrosine metabolism	ko00350	27	p-Coumaric acid
	Folate biosynthesis	ko00790	3	4-Hydroxybenzoic acid
	Phenylpropanoid biosynthesis	ko00940	66	p-Coumaric acid; Syringin; Scopoletin; Sinapyl alcohol
	Flavonoid biosynthesis	ko00941	12	L-Epicatechin
	Isoquinoline alkaloid biosynthesis	ko00950	10	p-Coumaric acid
GFP vs FP	Ubiquinone and other terpenoid-quinone biosynthesis	ko00130	8	4-Hydroxybenzoic acid
	Folate biosynthesis	ko00790	2	4-Hydroxybenzoic acid
	Phenylpropanoid biosynthesis	ko00940	69	Syringin; Caffeic aldehyde; Scopoletin; Sinapyl alcohol
FS vs FP	Ubiquinone and other terpenoid-quinone biosynthesis	ko00130	13	p-Coumaric acid
	Tyrosine metabolism	ko00350	7	p-Coumaric acid
	Phenylpropanoid biosynthesis	ko00940	34	Caffeic aldehyde; p-Coumaric acid
	Flavonoid biosynthesis	ko00941	11	L-Epicatechin
	Flavone and flavonol biosynthesis	ko00944	3	Isotrifoliin; 3,7-Di-O-methylquercetin; Syringetin
	Isoquinoline alkaloid biosynthesis	ko00950	4	p-Coumaric acid

Note: ko indicates KEGG Ontology.

## Discussion

### Localization of active components in different parts of *Gardenia jasminoides* fruit

*G*. *jasminoides* fruits are widely used in Asian countries to obtain natural colorants and in traditional Chinese herbal medicine since they exhibit homeostatic, hepatoprotective, analgesic, antiphlogistic, antipyretic, and hypolipidemic effects [44]. Studies have shown that iridoids and crocins are the main active substances in *G*. *jasminoides* fruit and show high medicinal value [45]. As iridoid glycosides such as geniposide and gardenoside are the main components of *G*. *jasminoides* fruit, their pharmacological activity and biosynthesis have been reported [44]. Studies on the biosynthesis pathways of iridoids from *G*. *jasminoides* have been conducted by Ye et al. [10]. The genes involved in iridoid biosynthesis were expressed mainly in flowers and fruits, whereas iridoids, such as gardenoside and geniposide, accumulated mainly in fruits. In this research, we selected the main iridoid glycoside organ of accumulation, the fruit, and divided it into pericarp and pulp sample to conduct transcriptome and metabolomics studies. In the present work, using the widely targeted metabolomics approach, the obtained ion abundance results showed that geniposide and shanzhiside methyl ester accumulated mainly in the sarcocarp of green fruit, gardenoside accumulated mainly in the sarcocarp of red fruit, and the other detected iridoid glycosides accumulated mainly in the peel of red fruit, indicating that the distribution of different iridoids differed among the organs. Additionally, crocins has been found in the stigmas of *Crocus sativus L*. and the fruit of *G*. *jasminoides* [21, 46, 47]. Because of the complex harvesting process and low yield of crocins, researchers have focused on *G*. *jasminoides* crocins. A transcriptome analysis of the leaves, green fruits, and red fruits of *G*. *jasminoides* was recently conducted to identify and predict the genes that encode key enzymes responsible for crocins production via comparison with *Crocus sativus L*. [21]. Our results showed that crocins mainly accumulated in the peel and sarcocarp of red fruits and that the ion abundance in green fruits was significantly lower than that in red fruits. Understanding the distribution pattern of active ingredients in different parts of Gardenia fruits will provide a theoretical basis for our follow up screening of fine germplasms and for understanding the molecular mechanisms of active ingredient synthesis.

### Characterization of the iridoids biosynthetic pathway

The developing technologies for largescale metabolite identification and transcriptome sequencing have made metabolomics a powerful tool for investigating the biological processes and active ingredients involved in plant development [48, 49]. Because of the pharmacological and economic importance of iridoids, there has been intense interest in understanding their biosynthetic mechanisms to develop biotechnological approaches for their targeted production [50]. In a previous transcriptomic and metabonomic study, researchers identified several unigenes bearing high sequence similarity to candidate genes for crocin and iridoid biosynthesis [10, 12, 20, 44]. The upstream MVA and MEP pathways produce early precursors of iridoid. In our study, the expression of *DXS* and *DXR* was found to vary, which indicated that there were multiple copies of the genes that regulate these enzymes, and their upstream regulation did not shown consistent changes. Therefore, future work is needed to isolate and clone the genes regulating these enzymes and to explore the function of the genes. In the iridoid pathway, *GPPS*, *GGPPS* and *GES* catalyze the synthesis of geraniol from geranyl pyrophosphate; geraniol is then transformed into 10-oxogeranial catalyzed by *GES*, *G10H* and *10-HGO*, and the transformation of iridotrial into downstream substances carried out. In our study, the genes encoding the enzymes of the G8O pathway downstream showed high expression level in green fruits, and their expression was higher in the sarcocarp than in the peel. According to our metabolomics results, we hypothesized that a transport mechanism existed in the peel and sarcocarp of *G*. *jasminoides* through which precursor substances generated in the sarcocarp were transported to the peel to initiate subsequent synthesis.

### A negative correlation between the biosynthesis of geniposide and crocin

Combined with the results of recent studies, we successfully constructed a metabolic map of the main active components of *G*. *jasminoides* and divided it into 5 pathways. Iridoids are derived from terpenoids, which are synthesized via the upstream MVA and MEP pathways and by the related enzymes in the iridoid pathway. The other portions of this map corresponded to crocin biosynthesis, including the upstream MEP pathway, the midstream carotenoid pathway, and the downstream crocin pathway. In the MVA and MEP pathways, *HMGS-1*, *FPPS-1*, *FPPS-2*, *DXS-3*, and *DXR-1* were shown lower expression level with the development of fruit, while other candidate unigenes were increased. In the iridoid pathway, in addition to G10H-1 and G10H-2, other unigenes were shown high expression level when the fruit became red. Additionally, *G80H-1*, *G80H-2*, were shown high expression level in the sarcocarp of the green fruit, which showed the same trend as the ion abundance of geniposide. As the fruit develops, the genes related to the synthesis of crocins were shown higher expression level. Additionally, the change trend of key enzyme genes involved in the crocin pathway was positively correlated with the change in the ion abundance of crocins. In particular, the expression levels and ion abundance in the peel were both high. In summary, candidate genes showed different expression trends during fruit development. When the fruits were young (green), iridoid biosynthesis dominated the metabolic pathways. High levels of iridoids, especially geniposide, are synthesized in the fruit. When the fruit turns red, key enzyme encoding genes related to the biosynthesis of crocin were shown higher expression level, leading to a significant increase in the ion abundance of crocins in the sarcocarp. It was demonstrated that different active ingredients and genes exhibit fruit tissue specific accumulation and expression in fruit. The redirection of metabolic flux and regulation of key enzymes may be the main reasons for the changes in the biosynthesis of iridoids and crocins in *G*. *jasminoides* fruit.

## Conclusion

This research is a first attempt to elucidate the whole biosynthetic pathway of iridoids and crocins in *G*. *jasminoides* fruit based on transcriptomic and metabolomic analysis. The key genes involved in iridoid and crocin biosynthesis, including *DXS*, *DXR*, *GES*, *IS*, *G8O*, *CCDs* and *UGTs*, were differentially expressed in different developmental stages and parts of fruits. DEGs and DAMs showed a negative correlation between the biosynthesis of geniposide and crocin. The redirection of the metabolic flux and the regulation of key enzymes may be the main reasons for the changes in the biosynthesis of iridoid and crocin in fruit. These researches effectively expanded the functional genomic library and provide new insights into iridoid and crocin biosynthesis in *G*. *jasminoides*.

## Supporting information

S1 Fig Validation of the transcription levels for selected DEGs by qRT-PCR. (TIF)

S1 Table Primer sequence for qRT-PCR. (XLSX)

S2 Table The distribution of splicing length. (XLSX)

S3 Table KEGG enrichment analysis of differential expression genes related to iridoid and crocin biosynthesis. (XLSX)

S4 Table The FPKM of the 24 selected genes. (XLSX)

S5 Table The FPKM of the 18 selected genes. (XLSX)

S6 Table Ion abundance of metabolites in samples. (XLSX)

## Abbreviations

10-HGO: 10-hydroxygeraniol oxidoreductase
ALDH: acetaldehyde dehydrogenase
CCD: carotenoid cleavage dioxygenase
CRTISO: carotenoid isomerase
DAM: differentially accumulated metabolite
DEG: differentially expression gene
DXR: 2-C-methyl-D-erythritol-4-phosphate reductoisomerase
DXS: 1-deoxy-D-xylulose-5-phosphate synthase
FP: the peel of red fruit
FPPS: farnesyl diphosphate synthase
FS: the sarcocarp of red fruit
G10H: geraniol 10-hydroxylase
G8O: 8-hydroxygeraniol dehydrogenase
GES: geraniol synthase
GFP: the peel of green fruit
GFS: the sarcocarp of green fruit
GGPPS: geranylgeranyl diphosphate synthase
GPPS: geranyl diphosphate synthase
HDR: 4-hydroxy-3-methyl-2-(E)-butenlyl diphosphate reductase
HMGR: 3-hydroxy-3-methyl glutaryl CoA reductase
HMGS: 3-hydroxy-3-methylglutaryl CoA synthase
IS: iridoid synthase
LCYB: lycopene cyclase
MK: mevalonate kinase
NCED: 9-cis-epoxycarotenoid dioxygenase
PDS: phytoene desaturase
PSY: phytoene synthase
SS: squalene synthase
UGTs: glucosyltransferases
ZDS: zeta-carotene desaturase
Z-ISO: zeta-carotene isomerase

## References

[1] Xiao W.P.; Li S.M.; Wang S.Y.; Ho C.T. Chemistry and bioactivity of *Gardenia jasminoides*. *J*. *Food Drug Anal*. 2017, 25, 43–61. doi: 10.1016/j.jfda.2016.11.005 28911543 PMC9333430

[2] Zhou Y.Y.; Zhang J.; Tang R.C.; Zhang J. Simultaneous dyeing and functionalization of silk with three natural yellow dyes. *Indus Crops Prod*. 2015, 64, 224–32.

[3] Hong K.; Jeon H.; Lee S. Extraction of natural dye from Gardenia and chromaticity analysis according to chiparameter. *J Ind Eng Chem*. 2015, 24, 326–32.

[4] Lim H.; Park KR.; Lee DU.; Kim YS.; Kim HP. Effects of the constituents of Gardenia Fructus on prostaglandin and NO reduction. *Biomol Ther*. 2008, 16, 82–86.

[5] Wu S.Y.; Wang G.F.; Liu Z.Q.; Rao J.J.; Lv L.; Xu W.; et al. Effect of geniposide, a hypoglycemic glucoside, on hepatic regulating enzymes in diabetic mice induced by a high fat diet and streptozotocin. *Acta Pharmacol Sin*. 2009, 30, 202–208. doi: 10.1038/aps.2008.17 19122671 PMC4002460

[6] Tao W.; Zhang H.; Xue W.; Ren L.; Xia B.; Zhou X.; et al. Optimization of supercritical fluid extraction of oil from the *Gardenia jasminoides* and its antidepressant activity. *Molecules* 2014, 19, 350–360. doi: 10.3390/molecules191219350 25429560 PMC6271100

[7] Chen Y.; Zhang H.; Li Y.X.; Huang J.; Zhao C.; Lin J.; et al. Crocin and geniposide profiles and radical scavenging activity of gardenia fruits (Gardenia jasminoides Ellis) from different cultivars and at the various stages of maturation[J]. *Fitoterapia* 2009, 81, 269–273. doi: 10.1016/j.fitote.2009.09.011 19815056

[8] Debnath T.; Park P.J.; Nath N.C.D.; Samad N.B.; Park H.W.; Lim B.O. Antioxidant activity of Gardenia jasminoides Ellis fruit extracts. *Food Chem*. 2011, 128, 697–703.

[9] Kuratsune H.; Umigai N.; Takeno R.; Kajimoto Y.; Nakano T. Effect of crocetin from Gardenia jasminoides Ellis on sleep: a pilot study. *Phytomedicine* 2010, 17, 840–843. doi: 10.1016/j.phymed.2010.03.025 20537515

[10] Ye P.; Liang S.C.; Wang X.M.; Duan L.X.; Jiang Y. F.Y; Yang J.F.; et al. Transcriptome analysis and targeted metabolic profiling for pathway elucidation and identification of a geraniol synthase involved in iridoid biosynthesis from Gardenia jasminoides. *Industrial Crops & Products* 2019, 132, 48–58.

[11] Nagatoshi M.; Terasaka K.; Nagatsu A.; Mizukami H. Iridoid specific glucosyltransferase from Gardenia jasminoides. *J*. *Biol*. *Chem*. 2011, 286, 32866–32874. doi: 10.1074/jbc.M111.242586 21799001 PMC3173207

[12] Ji A.J.; Jia J; Xu Z.C.; Li Y.; Bi W.; Ren F.M.; et al. Transcriptome Guided Mining of Genes Involved in Crocin Biosynthesis. *Front*. *Plant Sci*. 2017, 8, 518. doi: 10.3389/fpls.2017.00518 28443112 PMC5387100

[13] Vincent B.; Andrey O.; Martine C.; Marc R.; Benoit S. Co-expression of three MEP pathway genes and geraniol 10-hydroxylase in internal phloem parenchyma of Catharanthus roseus implicates multicellular translocation of intermediates during the biosynthesis of monoterpene indole alkaloids and isoprenoid derived primary metabolites. *Plant J* 2004, 38, 131–141. doi: 10.1111/j.1365-313X.2004.02030.x 15053766

[14] Alagna F.; Geu Flores F.; Kries H.; Panara F.; Baldoni L.; O’Connor S.; et al. Identification and Characterization of the Iridoid Synthase Involved in Oleuropein Biosynthesis in Olive (Olea europaea) Fruits. *J Biol Chem*. 2016, 291, 1–26. 26709230 10.1074/jbc.M115.701276 PMC4786697

[15] Loke K.K.; Rahnamaie Tajadod R.; Yeoh C.C.; Goh H.H.; Mohamed Hussein Z.A.; Zainal Z.; et al. Transcriptome analysis of Polygonumminus reveals candidate genes involved in important secondary metabolic pathways of phenylpropanoids and flavonoids. *Peer*J. 2017, 5, e2938. doi: 10.7717/peerj.2938 28265493 PMC5333554

[16] Liu Y.; Wang Y.; Guo F.X.; Zhan L.; Mohr T.; Cheng P.; et al. Deep sequencing and transcriptome analyses to identify genes involved in secoiridoid biosynthesis in the Tibetan medicinal plant Swertia mussotii. *Sci*. *Rep*.2017, 7, 43108. doi: 10.1038/srep43108 28225035 PMC5320516

[17] Pandey A.; Swarnkar V.; Pandey T.; Srivastava P.; Kanojiya S.; Mishra D.K.; et al. Transcriptome and Metabolite analysis reveal candidate genes of the cardiac glycoside biosynthetic pathway from Calotropis procera. *Sci*. *Rep*.2016, 6, 34464. doi: 10.1038/srep34464 27703261 PMC5050527

[18] Vashisht I.; Pal T.; Sood H.; Chauhan R.S. Comparative transcriptome analysis in different tissues of a medicinal herb, Picrorhiza kurroa pinpoints transcription factors regulating picrosides biosynthesis. *Mol*. *Biol*. *Rep*. 2016, 43, 1395–1409. doi: 10.1007/s11033-016-4073-0 27633652

[19] Rai A.; Kamochi H.; Suzuki H.; Nakamura M.; Takahashi H.; Hatada T.; et al. De novo transcriptome assembly and characterization of nine tissues of Lonicera japonica to identify potential candidate genes involved in chlorogenic acid, luteolosides, and secoiridoid biosynthesis pathways. *J*. *Nat*. *Med*. 2017, 71, 1–15. doi: 10.1007/s11418-016-1041-x 27629269 PMC5214891

[20] Mai N.; Kazuyoshi T.; Miki O.; Makiko S.; Tatsunori I.; Akito N.; et al. UGT75L6 and UGT94E5 mediate sequential glucosylation of crocetin to crocin in Gardenia jasminoides [J]. *FEBS Letters*, 2012, 586, 1055–1061. doi: 10.1016/j.febslet.2012.03.003 22569263

[21] Frusciante S.; Diretto G.; Bruno M.; Ferrante P.; Pietrella M.; Prado Cabrero A. Novel carotenoid cleavage dioxygenase catalyzes the first dedicated step in saffron crocin biosynthesis. *Proc*. *Natl*. *Acad*. *Sci*. 2014, 111, 12246–12251. doi: 10.1073/pnas.1404629111 25097262 PMC4143034

[22] Nisar N.; Li L.; Lu S.; Khin N.C.; Pogson B.J. Carotenoid metabolism in plants. *Mol*. *Plant*. 2015, 8, 68–82. doi: 10.1016/j.molp.2014.12.007 25578273

[23] Xu Z.; Peters R. J.; Weirather J.; Luo H.; Liao B.; Zhang X. Full length transcriptome sequences and splice variants obtained by a combination of sequencing platforms applied to different root tissues of Salvia miltiorrhiza and tanshinone biosynthesis. *Plant J*.2015, 82, 951–961. doi: 10.1111/tpj.12865 25912611

[24] Xu H.; Song J.; Luo H.; Zhang Y.; Li Q.; Zhu Y.; et al. Analysis of the genome sequence of the medicinal plant Salvia miltiorrhiza. *Mol*. *Plant*. 2016, 9, 949–952. doi: 10.1016/j.molp.2016.03.010 27018390 PMC5517341

[25] Xu Z.C.; Ji A.J.; Zhang X.; Song J.Y.; Chen S.L. Biosynthesis and regulation of active compounds in medicinal model plant Salvia miltiorrhiza. *Chin*. *Herb*. *Med*.2016, 8, 3–11.

[26] Xu Z.C.; Luo H.M.; Ji A.J.; Zhang X.; Song J.Y.; Chen S.L. Global identification of the full length transcripts and alternative splicing related to phenolic acid biosynthetic genes in Salvia miltiorrhiza. *Front*. *Plant Sci*. 2016, 7:100. doi: 10.3389/fpls.2016.00100 26904067 PMC4742575

[27] Ahrazem O.; Rubio Moraga A.; López R.C.; Gómez Gómez L. The expression of a chromoplast specific lycopene beta cyclase gene is involved in the high production of saffron’s apocarotenoid precursors. *J*. *Exp*. *Bot*. 2009, 61,105–119. 10.1093/jxb/erp283 19767307

[28] Zhang Z.; Tian C.P.; Zhang Y.; Li C.Z.Y.; Li X.; Qiang Y.; et al. Transcriptomic and metabolomic analysis provides insights into anthocyanin and procyanidin accumulation in pear. *BMC Plant Biol*.2020,20, 129. doi: 10.1186/s12870-020-02344-0 32220242 PMC7099803

[29] Yuan H.; Zeng X.; Shi J.; Xu Q.; Wang Y.; Jabu D. Time course comparative metabolite profiling under osmotic stress in tolerant and sensitive Tibetian hulless barley. *BioMed Research International*. 2018; 9415409: 1–12. 10.1155/2018/9415409 PMC6323448 30671479

[30] Wang Z., Cui Y.Y., Vainstein A., Chen S.W., Ma H.Q. Regulation of fig (Ficus carica L.) fruit color: metabolomic and transcriptomic analyses of the flavonoid biosynthetic pathway. *Front*. *Plant Sci*. 2017, 8, 1990. doi: 10.3389/fpls.2017.01990 29209349 PMC5701927

[31] Chen W.; Gong L.; Guo Z.; Wang W.S.; Zhang H.Y.; Liu X.Q.; et al. A novel integrated method for largescale detection, identification, and quantification of widely targeted metabolites: Application in the study of rice metabolomics. *Mol*. *Plant* 2013, 6, 1769–1780. doi: 10.1093/mp/sst080 23702596

[32] Zhu G.T.; Wang S.C.; Huang Z.J.; Zhang S.B.; Liao Q.G.; Zhang C.Z.; et al. Rewiring of the fruit metabolome in tomato breeding. *Cell* 2018, 172, 249–261. doi: 10.1016/j.cell.2017.12.019 29328914

[33] Yuan H.; Zeng X.; Shi J.; Xu Q.; Wang Y.; Jabu D. Time course comparative metabolite profiling under osmotic stress in tolerant and sensitive Tibetian hulless barley. *BioMed Research International*. 2018, 9415409, 1–12. 10.1155/2018/9415409 PMC6323448 30671479

[34] Grabherr M. G.; Haas B.J.; Yassour M. Full length transcriptome assembly from RNA-Seq data without a reference genome. *Nature Biotechnology*. 2011, 29, 644–652. doi: 10.1038/nbt.1883 21572440 PMC3571712

[35] Trapnell C.; Williams B.A.; Pertea G. Transcript assembly and quantification by RNA-Seq reveals unannotated transcripts and isoform switching during cell differentiation. *Nat Biotech*. 2010, 28, 511–515. doi: 10.1038/nbt.1621 20436464 PMC3146043

[36] Zhou K.; liu X.D.; Zhang D.; Yang Q.; Fu S.D.; Meng D.L.; et al. Flavonoid Biosynthesis Is Likely More Susceptible to Elevation and Tree Age Than Other Branch Pathways Involved in Phenylpropanoid Biosynthesis in Ginkgo Leaves[J]. *Front*. *Plant Sci*. 10:983. doi: 10.3389/fpls.2019.00983 31417595 PMC6682722

[37] Altschul S. F., Madden T. L., Schaffer A. A., Zhang J., Zhang Z., Miller W., et al. Gapped BLAST and PSI-BLAST: a new generation of protein database search programs. *Nucleic Acids Res*. 1997, 25, 3389–3402. doi: 10.1093/nar/25.17.3389 9254694 PMC146917

[38] Buchfink B., Xie C., and Huson D. H. Fast and sensitive protein alignment using DIAMOND. *Nat*. *Methods*. 2015, 12, 59–60. doi: 10.1038/nmeth.3176 25402007

[39] Finn R. D., Tate J., Mistry J., Coggill P. C., Sammut S. J., Hotz H. R., et al. The Pfam protein families database. *Nucleic Acids Res*. 2008, 36, D281–D288 doi: 10.1093/nar/gkm960 18039703 PMC2238907

[40] Gotz S., Garcia-Gomez J. M., Terol J., Williams T. D., Nagaraj S. H., Nueda M. J., et al. High-throughput functional annotation and data mining with the Blast2GO suite. *Nucleic Acids Res*. 2008, 36, 3420–3435. doi: 10.1093/nar/gkn176 18445632 PMC2425479

[41] Mao X, Cai T, Olyarchuk JG, Wei L. Automated genome annotation and pathway identification using the KEGG Orthology (KO) as a controlled vocabulary. Bioinformatics. 2005, 21(19):3787–93 doi: 10.1093/bioinformatics/bti430 15817693

[42] Haas B. J., Papanicolaou A., Yassour M., Grabherr M., Blood P. D., and Bowden J. De novo transcript sequence reconstruction from RNA-seq using the Trinity platform for reference generation and analysis. Nat. Protoc. 2013, 8, 1494–1512. doi: 10.1038/nprot.2013.084 23845962 PMC3875132

[43] Li B., and Dewey C. N. RSEM: accurate transcript quantification from RNA-Seq data with or without a reference genome. BMC Bioinformatics. 2011, 12:323. doi: 10.1186/1471-2105-12-323 21816040 PMC3163565

[44] Gao L.; Zhu B.Y. The Accumulation of Crocin and Geniposide and Transcripts of Phytoene Synthase during Maturation of Gardenia jasminoides Fruit. *Evidence Based Complementary and Alternative Medicine*, 2013, 686351, 1–6. doi: 10.1155/2013/686351 23634173 PMC3619689

[45] Nagatoshi M.; Terasaka K.; Nagatsu A.; Mizukami H. Iridoid specific Glucosyltransferase from Gardenia jasminoides. *J Biol Chem*. 2011, 286, 32866–32874. doi: 10.1074/jbc.M111.242586 21799001 PMC3173207

[46] Sheu S.J.; Hsin W.C. HPLC separation of the major constituents of Gardeniae fructus. J. High Resolut. *Chromatogr*. 21, 523–526.

[47] Xu Z.C.; Pu X.D.; Gao R.R.; Demurtas O.C.; Steven J.; Michaela R.; et al. Tandem gene duplications drive divergent evolution of caffeine and crocin biosynthetic pathways in plants. *BMC Biol*. 2020, 18, 63. doi: 10.1186/s12915-020-00795-3 32552824 PMC7302004

[48] Khan N.; Bano A.; Rahman M.A.; Rathinasabapathi B.; Babar M.A. UPLC-HRMS based untargeted metabolic profiling reveals changes in chickpea (Cicer arietinum) metabolome following long term drought stress. *Plant Cell Environ*. 2019, 42, 115–132. doi: 10.1111/pce.13195 29532945 PMC7379973

[49] Xu J.; Yan J.; Li W.; Wang Q.; Wang C.; Guo J.; et al. Integrative Analyses of Widely Targeted Metabolic Profiling and Transcriptome Data Reveals Molecular Insight into Metabolomic Variations during Apple (Malus domestica) Fruit Development and Ripening. *Int J Mol Sci*. 2020, 21, 4797. doi: 10.3390/ijms21134797 32645908 PMC7370097

[50] Vonny S.; Fang Y.; Joaquin A.; Vincenzo D. L. Virus induced gene silencing identifies Catharanthus roseus 7-deoxyloganic acid-7-hydroxylase, a step in iridoid and monoterpene indole alkaloid biosynthesis. *Plant J*. 2013, 76, 754–765. doi: 10.1111/tpj.12330 24103035

