# Supplementary figures and images for: Metabolomics integrated with transcriptomics reveals the distribution of iridoid and crocin metabolic flux in Gardenia jasminoides Ellis
Source: PLoS One. 2021 Sep 10;16(9):e0256802. doi: 10.1371/journal.pone.0256802 (PMC8432746; doi:10.1371/journal.pone.0256802)

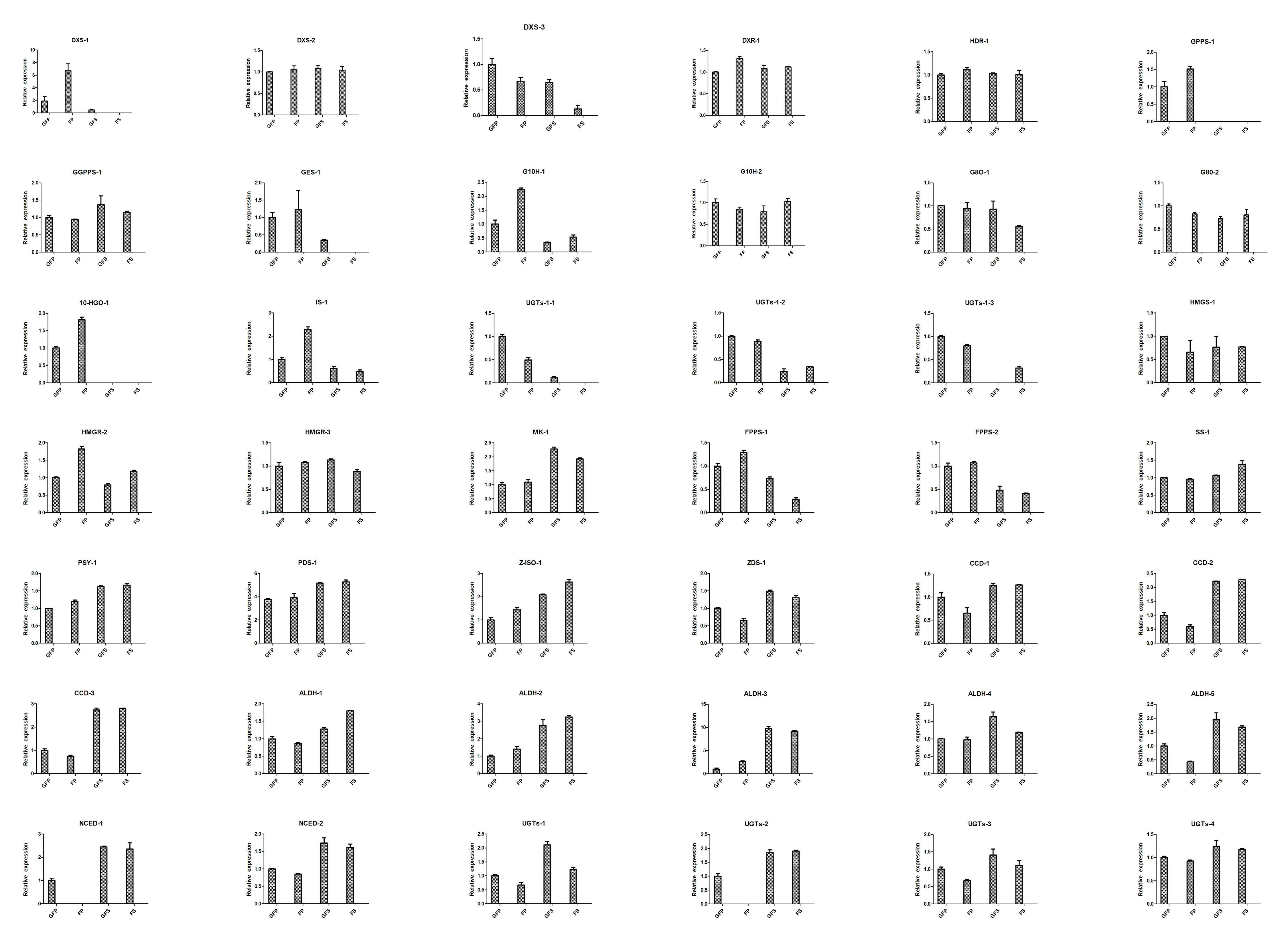

Supplement: S1 Fig — (TIF) [file pone.0256802.s001.tif]
